# *In vitro* Regeneration of Clematis Plants in the Nikita Botanical Garden via Somatic Embryogenesis and Organogenesis

**DOI:** 10.3389/fpls.2021.541171

**Published:** 2021-03-12

**Authors:** Irina Mitrofanova, Natalia Ivanova, Tatyana Kuzmina, Olga Mitrofanova, Natalya Zubkova

**Affiliations:** ^1^Plant Biotechnology and Virology Laboratory, Plant Developmental Biology, Biotechnology and Biosafety Department, FSFIS “The Nikita Botanical Gardens – National Scientific Center of the RAS,” Yalta, Russia; ^2^Structural Botany and Plant Reproductive Biology Section, FSFIS “The Nikita Botanical Gardens – National Scientific Center of the RAS,” Yalta, Russia; ^3^Floriculture Laboratory, FSFIS “The Nikita Botanical Gardens – National Scientific Center of the RAS,” Yalta, Russia

**Keywords:** somatic embryo, shoots regeneration, morphogenic capacity, plantlets, clematis

## Abstract

The effects of growth regulators, namely, 6-benzylaminopurine (BAP) and thidiazuron (TDZ), on the morphogenic capacity of 13 cultivars of clematis plants, in terms of their morphological structure formation, shoot regeneration, and somatic embryo development, are presented. The clematis cultivars ‘Alpinist,’ ‘Ay-Nor,’ ‘Bal Tsvetov,’ ‘Crimson Star,’ ‘Crystal Fountain,’ ‘Kosmicheskaya Melodiya,’ ‘Lesnaya Opera,’ ‘Madame Julia Correvon,’ ‘Nevesta,’ ‘Nikitsky Rosovyi,’ ‘Nikolay Rubtsov,’ ‘Serenada Kryma,’ and ‘Vechniy Zov’ were taken in collection plots of the Nikita Botanical Gardens for use in study. After explant sterilization with 70% ethanol (1 min), 0.3–0.4% Cl_2_ (15 min), and 1% thimerosal (10 min), 1-cm long segments with a single node were introduced to an *in vitro* culture. The explants were established on the basal MS medium supplemented with BAP (2.20–8.90 μM) and 0.049 μM NAA, or TDZ (3.0; 6.0, and 9.0 μM) with 30 g/L sucrose and 9 g/L agar. The medium with 0.89 μM BAP served as the control. Culture vessels and test tubes with the explants were maintained in plant growth chamber-controlled conditions: with a 16-h photoperiod, under cool-white light fluorescent lamps with a light intensity of 37.5 μmol m^–2^ s^–1^, at a temperature of 24 ± 1°C. Histological analysis demonstrated that adventitious bud and somatic embryo formation in studied clematis cultivars occurred at numerous areas of active meristematic cell zones. The main role of plant growth regulators and its concentrations were demonstrated. It was determined that maximum adventitious microshoot regeneration without any morphological abnormalities formed on the media supplemented with BAP or TDZ. 4.40 μM BAP, or 6.0 μM TDZ were optimal cytokinin concentrations for micropropagation. The explants of ‘Alpinist,’ ‘Ay-Nor,’ ‘Crimson Star,’ ‘Crystal Fountain,’ ‘Nevesta,’ and ‘Serenada Kryma’ cultivars displayed high morphogenetic capacity under *in vitro* culturing. During indirect somatic embryogenesis, light intensity 37.5 μmol m^–2^ s^–1^ stimulated a higher-number somatic embryo formation and a temperature of 26°C affected somatic embryo development. Active formation of primary and secondary somatic embryos was also demonstrated. 2.20 μM BAP with 0.09 μM IBA affected the high-number somatic embryo formation for eight cultivars. Secondary somatic embryogenesis by the same concentration of BAP was induced. The frequency of secondary somatic embryogenesis was higher in ‘Crystal Fountain’ (100%), ‘Crimson Star’ (100%), ‘Nevesta’ (97%), and ‘Ay-Nor’ (92%) cultivars. Based on these results, the methodology for direct somatic embryogenesis and organogenesis of studied clematis cultivars has been developed.

## Introduction

The genus *Clematis* L. belongs to the buttercup family (Ranunculaceae Juss.) and includes ca. 300 species and over 3000 cultivars (Surhone et al., 2011; Zubkova, 2015) of this mainly perennial flowering liana. Clematis plants are widely used in ornamental gardening, but many species are also of great economic importance, since they contain essential oils, tannins, vitamin C, and volatiles, and some may have fungicidal effects that inhibit the development of molds. Accordingly, certain clematis species are used in Tibetan, Chinese, and Mongolian medicine (Lloyd, 1989; Toomey and Leeds, 2001; Zubkova, 2015).

In the Nikita Botanical Gardens—National Scientific Center of the Russian Academy of Sciences (NBG-NSC), a collection of clematis plants has been created that includes 24 species and 236 cultivars of native and foreign origin (Zubkova, 2015). This valuable collection helps to conserve clematis species, yet also provides a variety of biomorphological features in one place for investigation, in addition to research into the breeding stages of these plants. The large-flowered clematis plants are propagated vegetatively because most hybrid cultivars lack viable seed progeny. Furthermore, many of the cultivated seedlings are often not decorative enough and fail to preserve the aesthetic features of the mother plant. Adding to this, clematis plants are significantly affected by viral, fungal, and bacterial diseases, which not only reduce their decorativeness but also limit their mass propagation. Over the past few years, a number of viral pathogens that cause various disease symptoms on the leaves and flowers of tested plants have been found in the NBG-NSC collection (Zakubanskiy et al., 2018a, b).

Conventional propagation methods are inefficient for obtaining sufficient amounts of both planting and raw material to meet the demand of the food and medical industries. Modern biotechnological methods, using such plant cell properties as totipotency, can ensure the successful multiplication of rare and single plants, as well as new cultivars and breeding forms. Depending on the species and cultivar used, however, morphogenetic capacity in plants can be realized in various ways. One of these is organogenesis, which is the process of *de novo* formation of adventitious shoots and roots from an unorganized growing callus mass (i.e., indirect regeneration) and directly from leaf, stem, germ, or flower cells (i.e., direct regeneration) (Pati et al., 2004; Pipino et al., 2008, 2010; Deepika and Kanwar, 2010). Another mode of plant regeneration is somatic embryogenesis, which is the process of asexual development of embryogenic structures from reproductive and somatic tissues in a way similar to zygotic embryogenesis (Mitrofanova, 2011; Nic-Can et al., 2015; Germanà and Lambardi, 2016; Martins et al., 2016; Loyola-Vargas and Ochoa-Alejo, 2018). In studying the plant regeneration features of *Clematis integrifolia* × *C. viticella*, no genetic changes in plants were obtained via somatic embryogenesis (Mandegaran and Sieber, 2000). Genome variability was established in regenerated clematis plants of the cultivar ‘Serenada Kryma,’ but this was dependent on direct and indirect somatic embryogenesis or organogenesis (Mitrofanova et al., 2003). Using ISSR primers, 105 amplicons were found of which six were polymorphic, with the heterogeneity of clematis plants averaging 5.7%, and this detected variability due to indirect organogenesis. In later work, a comparative study of morphogenetic capacity realization via direct somatic embryogenesis was carried out in eight clematis cultivars; this established the effects of biotic and abiotic factors upon somatic embryo formation and plant regeneration during cultivation (Mitrofanova et al., 2007; Mitrofanova, 2011). Indirect somatic embryogenesis has been described in the clematis cultivar ‘Multi-Blue,’ for which histological analysis confirmed the formation of embryogenic structures in its callus (Zhang et al., 2011). Earlier, anatomical research had confirmed the formation of morphogenic structures and adventitious roots as a result of organogenesis in the same cultivar (Zhang et al., 2010). Among clematis hybrids, a comparative histological study of embryogenic structures and zygotic embryos revealed differences in their *in vitro* development (Arene et al., 2006). Chlorophyll and anthocyanin content of microshoots in *C. pitcheri* Torr. & A. Gray was changed during its cultivation on culture media having different concentrations of nitrogen and sucrose under variable temperature conditions (Kawa-Miszczak et al., 2009). More recently, the biochemical and physiological characteristics of some clematis cultivars grown in the NBG-NSC open-field collection and cultured *in vitro* have been presented (Brailko et al., 2018), and the effect of ribavirin on clematis plant improvement was reported in Ivanova et al. (2018). The medicinal plants *C. gouriana* Roxb. and *C. heynei* M.A. Rau were successfully regenerated by direct and indirect organogenesis, with the resulting plants then planted for *ex vitro* acclimatization (Naika and Krishna, 2008; Chavan et al., 2012). Finally, the effects of various substrates on microshoots and softwood stem cuttings’ rooting were studied in five clematis cultivars from the *Atragene* section (Kreen et al., 2002).

In sum, as evinced by the foregoing, the morphogenetic capacity of certain clematis cultivars and species has been mainly studied, albeit to various extents. However, we still lack a thorough and robust evaluation of the regenerative capacity of clematis plants cultured *in vitro* under the combined influences from multiple factors, namely, genotype, plant growth regulators, light intensity, and temperature (among others). Therefore, this study’s main objective was to assess the morphogenetic potential of explants from 13 clematis cultivars, under the influence of various culture factors, at stages of formation induction of morphogenic structures and plant regeneration. The obtained results could be used for the subsequent propagation of healthy plants and the creation of an *in vitro* gene bank.

## Materials and Methods

### Plant Material and Culture Establishment

The mother plants of 13 clematis cultivars—‘Alpinist,’ ‘Ay-Nor,’ ‘Bal Tsvetov,’ ‘Crimson Star,’ ‘Crystal Fountain,’ ‘Kosmicheskaya Melodiya,’ ‘Lesnaya Opera,’ ‘Madame Julia Correvon,’ ‘Nevesta,’ ‘Nikitsky Rosovyi,’ ‘Nikolay Rubtsov,’ ‘Serenada Kryma,’ and ‘Vechniy Zov’—grown at the collection plot of ornamental plants in the Nikita Botanical Gardens (Yalta, Russian Federation) were used as explant sources (Table 1).

**TABLE 1:** List of investigated clematis cultivars.

Cultivar	Garden group	Originator and main characteristics
Alpinist	Lanuginosa	M.A. Beskaravaynaya, 1974. Shrubby climber up to 2.5–3.5 m long. Leaves are compound, with 5 leaflets, green and light green. Flowers are lilac white, 10.0–14.0 cm in diameter; anthers are yellow. Remontant cultivar of late flowering (II decade of July). Pruning group #2.
Ay-Nor	Viticella	M.A. Beskaravaynaya, 1972. Shrubby climber up to 2.0 m long. Leaves are compound, ternate, rarely simple, dark green. Flowers are pink, with a blue-purple tinge at the base, 12.0–14.0 cm in diameter; anthers are yellow. Remontant cultivar with the middle terms flowering (III decade of May). Pruning group #3.
Bal Tsvetov	Lanuginosa	M.A. Beskaravaynaya, 1972. Shrubby climber up to 2.0 m long. Leaves are compound, ternate, dark green. Flowers are violet-blue with a violet-purple stripe in the center of the sepals, 15.0–18.0 cm in diameter; anthers are brown. Remontant cultivar of early flowering (II decade of May). Pruning group #2.
Crimson Star	Lanuginosa	Shrubby climber up to 2.0–2.5 m long. Leaves are ternate, green. Flowers are crimson red, 10.0–12.0 cm in diameter; anthers are yellow. Remontant cultivar of early flowering (II decade of May). Pruning group #2.
Crystal Fountain [syn. ‘Fairy Blue’]	Florida	H. Hayakawa, 1994. Shrubby climber up to 2.0 m long. Leaves are ternate, green. Flowers are purple-blue, pale blue in the center, double, 10.5–12.0 cm in diameter. Remontant cultivar of early flowering (II decade of May). Pruning group #2.
Kosmicheskaya Melodiya	Jackmanii	A.N. Volosenko-Valenis, M.A. Beskaravaynaya, 1965. Shrubby climber up to 3.0 m long. Leaves are compound, with 3–5 leaflets. Purple flowers, 10.0–14.0 cm. in diameter; anthers are of dark cherry color. Profusely and long blooming cultivar with the middle terms of flowering (III decade of May). Pruning group #3.
Lesnaya Opera	Viticella	M.A. Beskaravaynaya, 1972. Shrubby climber up to 2.5–2.8 m long. Leaves are compound, consisting of three green leaves. Flowers are white, 10.0–14.0 cm in diameter; anthers are yellow. Remontant cultivar with the middle terms of flowering (III decade of May). Pruning group #3.
Madame Julia Correvon	Viticella	F. Morel, 1900. Shrubby climber up to 2.2–3.0 m long. Leaves are unequally pinnate, the lower ones are often ternate, green. Flowers are red-purple, 10.5–12.5 cm in diameter; anthers are yellow. Profusely blooming remontant cultivar with the middle terms of flowering (III decade of May). Pruning group #3.
Nevesta	Lanuginosa	M.A. Beskaravaynaya, E.A. Donyushkina, 1979. Shrubby climber up to 3.0 m long. Leaves are simple and compound, consist of 1–3–5–7 green and light green leaves that burn in the sun. Flowers are grayish-white 13.0–15.0 cm in diameter; anthers are yellow. Remontant cultivar with the middle terms of flowering (I decade of June). Pruning group #2.
Nikitsky Rozovyi	Viticella	A.N. Volosenko-Valenis, M.A. Beskaravaynaya, 1965. Shrubby climber up to 2.1–2.8 m long. Leaves are ternate, green and dark green, not burn in the sun. Flowers are pink, 12.0–14.0 cm in diameter; anthers are light yellow. Remontant cultivar with the middle terms of flowering (II decade of June). Pruning group #3.
Nikolay Rubtsov	Jackmanii	A.N. Volosenko-Valenis, M.A. Beskaravaynaya, 1967. Shrubby climber up to 2.5 m long. Leaves are compound, with 3–5 leaflets, green. Pink flowers are lighter toward the center, 10.0–17.0 cm in diameter; anthers are light yellow. Remontant cultivar of early flowering (II decade of May). Pruning group #3.
Serenada Kryma	Lanuginosa	M.A. Beskaravaynaya, 1978. Shrubby climber up to 3.5 m long. Ternate leaves of dark green color. Flowers are violet-blue, with carmine veins and a light middle, 13.5–17.0 cm in diameter; anthers are brownish. Remontant cultivar of early flowering (II decade of May). Pruning group #2.
Vechniy Zov	Jackmanii	M.A. Beskaravaynaya, E.A. Donyushkina, 2003. Shrubby climber up to 2.5–3.0 m long. Leaves are compound, with 3–5 leaflets, green, and light green. Flowers are crimson-pink, 0.9–10.0 cm in diameter; anthers are light yellow. Remontant cultivar with the middle terms of flowering (III decade of May). Pruning group #3.

The investigations were carried out in the Laboratory of Plant Biotechnology and Virology of the Plant Developmental Biology, Biotechnology and Biosafety Department, Federal State Funded Institution of Science “The Nikita Botanical Gardens—National Scientific Center of the RAS.” To obtain an aseptic culture, plant material isolated in January–February 2017–2019 underwent sequential sterilization in this way: 1 min in 70% ethanol, followed by 15 min in 0.3–0.4% chlorine-containing solution (Dez Tab, China) and 10 min in 1% thimerosal solution (Sigma, United States) with 1–2 drops of Tween 20. After adding each reagent, the shoot segments 3 cm long were washed three times in sterile distilled water. After shoot segments had been surface-sterilized with a contamination frequency not greater than 10%, the nodal explants 1 cm long with axillary buds were established in test tubes on the MS (Murashige and Skoog, 1962) induction culture medium supplemented with 0.89 μM 6-benzylaminopurine (BAP, Sigma, United States), 30 g/L sucrose, and 9 g/L of agar (PanReac, Spain). For the elimination of viral infection, 10 mg/L virocid ribavirin (Virazole, 1-β-D-ribofuranosyl-1,2,4-triazole-3-carboxamide, Duchefa Biochemie, Holland) was added to the medium at the explant introduction stage. Subculturing was carried out at 2 week intervals. All plant handling and treatment were done under aseptic conditions in the SC2 laminar flow cabinet (ESCO, Singapore).

### Organogenesis Induction and Plant Regeneration

To induce organogenesis and plant regeneration, 1 cm long microshoots and microcuttings of 13 cultivars were used. Explants were placed on the MS culture medium with MS vitamins and various concentrations of plant growth regulators, 2.20–8.90 μM BAP in combination with 0.049 μM α-naphthylacetic acid (NAA, Duchefa Biochemie, Holland) or 3.0–9.0 μM thidiazuron (TDZ, Duchefa Biochemie, Holland), supplemented with 100 mg/L myo-inositol (Duchefa Biochemie, Holland), 30 g/L sucrose, and 9 g/L of agar. As the control, the MS medium with 0.89 μM BAP was used. Medium pH was 5.7–5.8 for all culture media, which were autoclaved at 120°C for 7–12 min in a LAC 5060S sterilizer (Daihan Labtech, South Korea). Plant growth regulators and vitamins were first sterilized by cold filtration through MILLEX^®^ GP filters (0.22 μm) and then added to the media after the autoclaving. Culture vessels (100 or 250 ml jars) with explants were maintained in “BIOTRON” growth chambers and in a plant growth chamber (MLR-352-PE, Panasonic, Japan) at 24 ± 1°C, with a 16-h photoperiod under cool-white light fluorescent lamps (Philips TL, 40 W: light intensity of 37.5 μmol m^–2^ s^–1^). Subculturing of each cultivar was carried out at 3–4 week intervals.

### Somatic Embryogenesis Induction and Plant Regeneration

For the induction experiments of indirect somatic embryogenesis, clematis microshoots or microcuttings with one or two internodes of 13 cultivars were cultured on a solidified MS medium with MS vitamins, 100 mg/L of myo-inositol, and different plant growth regulators. Firstly, microcuttings of 13 cultivars with one or two internodes were placed on an MS culture medium supplemented with 1.8 μM zeatin and 0.04 μM indole-3-butyric acid (IBA, Duchefa Biochemie, Holland) to induce callus formation. To induce embryogenic callus formation, 0.9–6.8 μM zeatin (Sigma, United States) or 0.9–6.8 μM 2,4-D (2,4-dichlorophenoxyacetic acid; Sigma, United States) was used. Callus was placed on an MS medium with 0.4–4.6 μM zeatin in order to induced indirect somatic embryogenesis. To induce the secondary somatic embryo formation, primary somatic embryos derived from callus were transferred to media with 0.4–4.6 μM IBA and 1.8 μM zeatin. All culture media were supplemented with 30 g/L sucrose, and the media were solidified with 9 g/L agar. The plant growth regulator-free medium served as the control. Culture vessels (100 or 250 ml jars) with microshoots, microcuttings, and somatic embryos were maintained at 24 ± 1°C, with a 16-h photoperiod under cool-white light fluorescent lamps (Philips TL, 40 W: 37.5 μmol m^–2^ s^–1^ light intensity). In an experiment with somatic embryogenesis induction, the culture vessels with calli were placed in growth chambers with temperature (20–30°C) and light intensity (5–60 μmol m^–2^ s^–1^) regulated under a 16-h photoperiod. Subculturing of each cultivar was carried out at 3–4 week intervals.

For the direct somatic embryogenesis induction experiments, firstly, vegetative buds of 13 clematis cultivars were cultured on an MS medium with 0.44–8.9 μM BAP and 0.09 μM IBA. The plant growth regulator-free medium served as the control. Due to the low rate of direct somatic embryogenesis induction in five cultivars, the main experiments with ‘Ay-Nor,’ ‘Crimson Star,’ ‘Crystal Fountain,’ ‘Kosmicheskaya Melodiya,’ ‘Lesnaya Opera,’ ‘Nevesta,’ ‘Serenada Kryma,’ and ‘Vechniy Zov’ cultivars were followed. For the secondary somatic embryogenesis induction, the primary somatic embryos were cultured on an MS medium with 0.89 or 2.22 μM BAP. All culture media were supplemented with 30 g/L sucrose and media solidified with 9 g/L agar. Cultures were incubated at 24 ± 1°C, 16-h photoperiod under cool-white light fluorescent lamps (Philips TL, 40 W: 37.5 μmol m^–2^ s^–1^ light intensity). Subculturing of each cultivar was carried out at 3–4 week intervals.

### Histological Analysis of Morphogenic Structures

The morphogenic structures that formed during organogenesis and somatic embryogenesis of clematis cultivars were subjected to histological analysis. Four cultivars with six regenerating structures from each (a total of 24 samples) were analyzed: ‘Alpinist’ and ‘Madame Julia Correvon’—bud conglomerates, ‘Crystal Fountain’—callus with somatic embryos, and ‘Nevesta’ and ‘Crystal Fountain’—somatic embryos on different stages. Slides for this analysis were prepared according to the commonly used methods (refer to Zhinkina and Voronova, 2000). Briefly, callus and somatic embryo conglomerates were fixed in formalin-aceto-alcohol (FAA) solution; after fixation, the material was transferred to a 70% ethyl alcohol solution. For material dehydration, isopropyl alcohol was used. Then, the material was maintained in two xylene solutions, for 2 h in each, and then embedded in paraffin. Infiltration with paraffin was performed over a 7 day period. Serial sections of callus and somatic embryo conglomerates were cut into the slides 10 μm thick with a rotary semiautomatic microtome RMD-3000 (MedTehnikaPoint, Russia) and affixed to permanent slides stained with methyl green–pyronin and alcian blue, as well as hematoxylin and alcian blue. The slides were analyzed under a light microscope (AxioScope A.1, Zeiss, Germany) using the bright-field method and polarized light observations. Microphotographs were taken with an AxioCam ERc 5s unit (Zeiss, Germany) and an IXUS 265HS digital camera (Canon Inc., Japan). To analyze the obtained images, AxioVisionRel v4.8.2 software (Zeiss, GmbH, Germany) was used.

### Statistical Analyses

Each treatment consisted of five glass vessels with four explants of each clematis cultivar (microshoots, microcuttings, callus, somatic embryos), repeated in triplicate. The number of vegetative buds regenerating the callus or somatic embryos was recorded after 2–4 weeks of culturing. The frequency of this regeneration was calculated as the average percentage of buds or calli which formed morphogenic structures. The number of microshoots per explant was determined on a monthly basis. All of the obtained data were processed in the Statistica for Windows program v10.0 (StatSoft, Inc., United States). The data of amount of regenerated microshoots per explant, length of explant, and number of internodes were analyzed, statistically averaging analysis of the variance (ANOVA) and standard deviation to find the variability between the different treatments. To see which means differed from each other and to indicate the significant difference during the analysis of percentage and number of investigated explants, Duncan’s multiple-range test was used (at *P* < 0.05).

## Results

### Organogenesis Induction and Plant Regeneration

**FIGURE 1:**
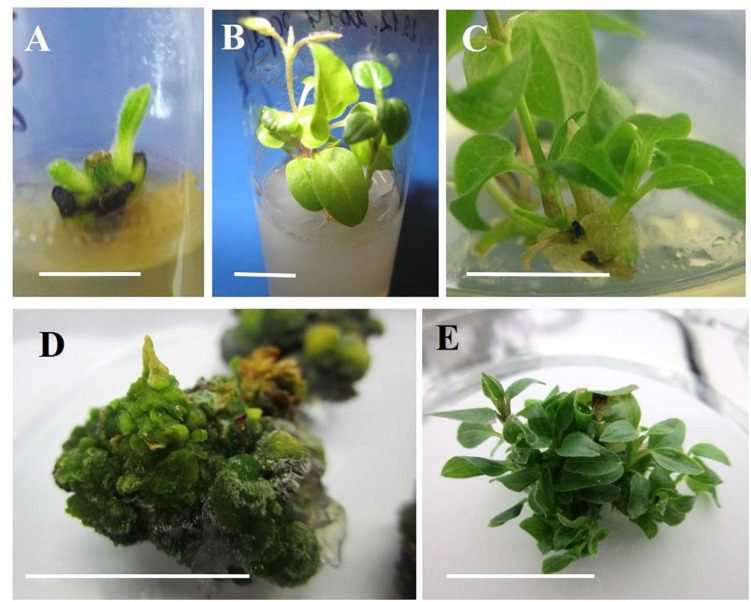
Indirect organogenesis in vegetative buds of clematis plant cultivars. **(A)** Vegetative bud development in the cultivar ‘Alpinist’ on the MS medium supplemented with 0.89 μM BAP and 10 mg/L ribavirin at the 1st week of culturing. **(B)** Single microshoots of the cultivar ‘Alpinist’ obtained on the same culture medium at 2–3 weeks after establishing the culture. **(C)** Callus formation on the base of a microshoot of the cultivar ‘Serenada Kryma’ on the medium with 4.40 μM BAP and 0.049 μM. **(D)** Compact morphogenic callus of cultivar ‘Crystal Fountain’ on the culture medium with 6 μM TDZ. **(E)** Conglomerate of the regenerated microshoots of cultivar ‘Crystal Fountain’ after 3–4 weeks of callus culturing with 6 μM TDZ. Scale bars correspond to 1 cm **(A–E)**.

The factors inducing explant development during *in vitro* culturing were the type and concentration of plant growth regulators in the culture medium. The initiation of vegetative bud development on the MS medium supplemented with 0.89 μM BAP and 10 mg/L ribavirin was observed within the 1st week of culturing in all 13 investigated cultivars (Figure 1A). Single microshoot formations were observed in the 2nd week of culturing, but at the base of these microshoots, the callus was poorly formed (Figures 1B,C). Both the obtained microshoots and calli were segmented after culturing on the MS medium with 2.20–4.40 μM BAP and 0.049 μM NAA, or the medium with 3.0–9.0 μM TDZ. In the case of BAP, it marginally increased the number of adventitious buds and microshoot regeneration. After 3–4 weeks of culturing, the best response in terms of the amount of morphogenic callus, adventitious buds, and microshoot formation was observed on the medium with TDZ (Figures 1D,E). A high number of regenerated shoots per explant on culture media supplemented with 6 and 9 μM TDZ were obtained (Table 2). Among the tested genotypes on the medium with 6 μM of TDZ, the cultivars ‘Alpinist,’ ‘Crystal Fountain’ and ‘Crimson Star’ regenerated seven microshoots per explant, while ‘Bal Tsvetov,’ Madame Julia Correvon,’ ‘Nikitsky Rosovyi,’ ‘Serenada Kryma,’ ‘Vechniy Zov’ developed more than four microshoots per explant. On the medium with 9 μM TDZ, in some cultivars, their number of adventitious microshoots increased to 11 per explant; however, some of these were hydrated. Therefore, for further micropropagation, 6 μM TDZ was used, which provided more adventitious microshoot regeneration. Using 4.40 μM BAP with 0.049 μM NAA in the experiments also promoted adventitious shoot formation in all the studied clematis cultivars. For example, 2.5 microshoots per explant were obtained for the cultivar ‘Alpinist,’ and likewise 2.4 microshoots per explant for ‘Crystal Fountain’ and 2.3 microshoots per explant for both ‘Nikitsky Rosovyi’ and ‘Madame Julia Correvon’ (Table 2).

**TABLE 2:** Regeneration responses of different clematis cultivars that were cultured on the MS medium with 6-benzylaminopurine (BAP) and 0.049 μM NAA, or thidiazuron (TDZ).

Cultivar*	Average number of regenerated microshoots per explant						
	BAP (μM)				TDZ (μM)		
	0.89**	2.2	4.4	8.9	3.0	6.0	9.0
A	0.7 ± 0.3^ab^	1.5 ± 0.9^a^	2.5 ± 1.1^a^	3.0 ± 1.2^a^	5.0 ± 1.4^a^	7.0 ± 1.6^a^	11.0 ± 1.9^a^
A-N	0.4 ± 0.1^de^	1.1 ± 0.2^ef^	1.9 ± 0.8^de^	2.3 ± 0.7^c^	2.4 ± 0.8^bc^	4.0 ± 1.8^bc^	5.0 ± 1.0^ef^
BT	0.4 ± 0.1^de^	1.2 ± 0.4^cd^	2.3 ± 1.2^ab^	2.7 ± 1.2^b^	2.3 ± 1.3^c^	4.2 ± 1.7^b^	6.0 ± 0.8^bc^
CS	0.6 ± 0.4^bc^	1.4 ± 0.6^ab^	2.3 ± 1.2^ab^	2.3 ± 1.1^c^	4.1 ± 1.7^a^	7.2 ± 1.6^a^	8.6 ± 1.2^ab^
CF	0.7 ± 0.2^b^	1.3 ± 0.9^bc^	2.4 ± 1.1^a^	2.8 ± 1.2^a^	5.0 ± 1.6^a^	7.0 ± 1.9^a^	9.0 ± 2.0^a^
KM	0.3 ± 0.1^f^	0.9 ± 0.6^gh^	1.4 ± 0.8^h^	2.1 ± 0.9^e^	1.8 ± 0.6^tf^	3.0 ± 1.8^fg^	4.4 ± 1.8^gh^
LO	0.4 ± 0.1^de^	0.7 ± 0.6^h^	1.2 ± 0.6^i^	2.2 ± 0.4^d^	1.6 ± 0.2^gh^	3.0 ± 1.8^fg^	4.4 ± 1.8^gh^
MJC	0.6 ± 0.3^cd^	1.4 ± 0.7^ab^	2.3 ± 1.3^ab^	2.7 ± 1.4^ab^	3.0 ± 1.3^ab^	4.0 ± 1.8^bc^	6.0 ± 1.0^bc^
N	0.4 ± 0.1^de^	1.4 ± 0.3^b^	2.1 ± 1.2^bc^	3.0 ± 1.2^a^	2.3 ± 1.3^c^	3.7 ± 1.3^de^	6.0 ± 0.8^bc^
NRo	0.6 ± 0.3^cd^	1.3 ± 0.9^bc^	2.2 ± 1.3^ab^	2.7 ± 1.2^b^	3.0 ± 1.3^ab^	4.0 ± 1.8^bc^	6.0 ± 1.0^bc^
NRu	0.4 ± 0.1^de^	1.6 ± 0.8^a^	2.2 ± 1.1^bc^	2.8 ± 0.9^a^	2.0 ± 0.6^d^	3.8 ± 0.9^cd^	8.6 ± 1.4^ab^
SK	0.7 ± 0.3^b^	1.4 ± 0.6^ab^	2.2 ± 1.1^bc^	2.6 ± 1.2^b^	2.8 ± 1.1^ab^	4.4 ± 1.8^ab^	6.7 ± 1.3^b^
VZ	0.8 ± 0.1^a^	1.1 ± 0.2^ef^	1.7 ± 0.3^h^	2.2 ± 0.4^d^	2.6 ± 1.2^b^	4.0 ± 1.8^bc^	5.8 ± 1.1^d^

*A, ‘Alpinist’; A-N, ‘Ay-Nor’; BT, ‘Bal Tsvetov’; CS, ‘Crimson Star’; CF, ‘Crystal Fountain’; KM, ‘Kosmicheskaya Melodiya’; LO, ‘Lesnaya Opera’; MJC, ‘Madame Julia Correvon’; N, ‘Nevesta’; NRo, ‘Nikitsky Rosovyi’; NRu, ‘Nikolay Rubtsov’; SK, ‘Serenada Kryma’; VZ, ‘Vechniy Zov.’ **Control culture medium. Different lowercase letters in the same column indicated the significant difference at P ≤ 0.05 (Duncan’s multiple-range test).

On the culture medium with 6 μM TDZ, the average length of each microshoot was 1.5 cm for the cultivars ‘Alpinist’ and ‘Crystal Fountain,’ 1.4 cm for ‘Nikitsky Rosovyi’ and ‘Madame Julia Correvon,’ and 1.3 cm for ‘Bal Tsvetov’ (Table 3). Correspondingly, on the culture medium supplemented with 9 μM TDZ, the number of internodes was 3.6–4.6 per microshoot; these microshoots were all well-formed, were compact, have shortened internodes, and have a bright-green color. Further subculturing increased the number of adventitious shoots. Thus, in clematis cultivars ‘Alpinist,’ ‘Crystal Fountain,’ ‘Madame Julia Correvon,’ and ‘Crimson Star,’ the number of shoots had reached 10–15 per explant after 4 weeks of culturing. The average number of internodes on culture media, supplemented with 2.20–8.90 μM BAP and 0.049 μM NAA, was 3.0–3.4 per microshoot. At the same time, some elongated internodes were noted, as well as the formation of single, disproportionately large leaves. The data presented in Table 3 demonstrates that adventitious microshoots of maximum length developed on the culture medium supplemented with 8.90 μM BAP and 0.049 μM NAA. Yet, higher BAP concentration stimulated the appearance of numerous morphological changes: the formation of strained shoots, hydration, twisting of leaves, the presence of a yellow-green color, and the formation of loose callus at the base of explants, all of which hindered their development and its pace.

**TABLE 3:** Morphometric characteristics (means ± SE) of adventitious microshoots in 13 clematis cultivars cultured on the MS medium with different concentrations of thidiazuron (TDZ) and 6-benzylaminopurine (BAP) with 0.049 μM NAA.

Cultivar	TDZ (μM)					
	3.0		6.0		9.0	
	Length of explant (cm)	Number of internodes	Length of explant (cm)	Number of internodes	Length of explant (cm)	Number of internodes
Alpinist	1.1 ± 0.01^a^	3.0^a^	1.5 ± 0.04^a^	4.0^a^	1.9 ± 0.04^a^	4.6 ± 0.2^a^
Ay-Nor	1.0 ± 0.01^ab^	2.0^b^	1.2 ± 0.01^bc^	3.0^bc^	1.6 ± 0.03^bc^	4.0^bc^
Bal Tsvetov	1.0 ± 0.03^ab^	3.0^a^	1.3 ± 0.04^b^	3.2 ± 0.3^ab^	1.8 ± 0.03^ab^	4.4 ± 0.4^ab^
Crimson Star’	0.9 ± 0.01^b^	2.0^b^	1.2 ± 0.01^bc^	3.0^bc^	1.6 ± 0.03^bc^	4.0^bc^
Crystal Fountain	1.1 ± 0.03^a^	3.0^a^	1.5 ± 0.04^a^	4.0^a^	1.8 ± 0.03^ab^	4.4 ± 0.4^ab^
Kosmicheskaya Melodiya	0.9 ± 0.01^b^	2.0^b^	1.2 ± 0.01^bc^	3.0^bc^	1.6 ± 0.03^bc^	4.0^bc^
Lesnaya Opera	1.0 ± 0.02^ab^	2.0^b^	1.2 ± 0.01^bc^	3.0^bc^	1.6 ± 0.03^bc^	4.0^bc^
Madame Julia Correvon	1.0 ± 0.02^ab^	3.0^a^	1.4 ± 0.03^ab^	3.2 ± 0.3^ab^	1.7 ± 0.04^b^	4.0^bc^
Nevesta	0.9 ± 0.01^b^	2.0^b^	1.2 ± 0.01^bc^	3.0^bc^	1.6 ± 0.03^bc^	4.0^bc^
Nikitsky Rosovyi	1.0 ± 0.04^ab^	3.0^a^	1.4 ± 0.02^ab^	3.2 ± 0.3^ab^	1.8 ± 0.04^ab^	4.0^bc^
Nikolay Rubtsov	0.8 ± 0.01^c^	2.0^b^	1.1 ± 0.01^de^	3.0^bc^	1.4 ± 0.02^de^	3.6 ± 0.2^cd^
Serenada Kryma	0.9 ± 0.01^b^	2.0^b^	1.2 ± 0.01^bc^	3.0^bc^	1.6 ± 0.03^bc^	4.0^bc^
Vechniy Zov	0.9 ± 0.01^b^	2.0^b^	1.2 ± 0.01^bc^	3.0^bc^	1.6 ± 0.03^bc^	4.0^bc^

	**BAP (μM)**					
	**2.2**		**4.4**		**8.9**	

Alpinist	1.08 ± 0.04^ab^	3.0^ab^	1.4 ± 0.03^a^	3.2 ± 0.2^a^	2.0 ± 0.04^a^	3.4 ± 0.2^ab^
Ay-Nor	0.86 ± 0.01^d^	2.0^bc^	1.0 ± 0.01^cd^	3.0^a^	1.4 ± 0.02^cd^	3.0^c^
Bal Tsvetov	1.20 ± 0.03^a^	3.0^ab^	1.2 ± 0.04^ab^	3.0^a^	1.9 ± 0.02^ab^	3.4 ± 0.1^ab^
Crimson Star’	0.90 ± 0.02^bc^	2.0^bc^	1.0 ± 0.01^cd^	3.0^a^	1.6 ± 0.02^bc^	4.0^a^
Crystal Fountain	1.10 ± 0.03^a^	3.0^ab^	1.3 ± 0.02^ab^	3.0^a^	1.7 ± 0.04^b^	3.0^c^
Kosmicheskaya Melodiya	0.96 ± 0.02^ab^	2.0^bc^	1.0 ± 0.01^cd^	3.0^a^	1.4 ± 0.02^cd^	3.0^c^
Lesnaya Opera	0.90 ± 0.02^bc^	2.0^bc^	1.2 ± 0.01^ab^	3.0^a^	1.7 ± 0.04^b^	4.0^a^
Madame Julia Correvon	0.98 ± 0.02^ab^	3.0^ab^	1.3 ± 0.03^ab^	3.0^a^	1.7 ± 0.04^b^	3.0^c^
Nevesta	0.96 ± 0.02^ab^	2.0^bc^	1.2 ± 0.01^ab^	3.0^a^	1.6 ± 0.02^bc^	3.0^c^
Nikitsky Rosovyi	1.06 ± 0.04^ab^	3.2 ± 0.2^a^	1.3 ± 0.02^ab^	3.0^a^	1.8 ± 0.04^ab^	3.2 ± 0.3^b^
Nikolay Rubtsov	0.90 ± 0.02^bc^	2.0^bc^	1.1 ± 0.01^bc^	3.0^a^	1.7 ± 0.04^b^	4.0^a^
Serenada Kryma	0.88 ± 0.01^c^	2.0^bc^	1.0 ± 0.01^cd^	3.0^a^	1.9 ± 0.02^ab^	4.0^a^
Vechniy Zov	0.98 ± 0.02^ab^	2.0^bc^	1.2 ± 0.01^ab^	3.0^a^	1.8 ± 0.04^ab^	4.0^a^

*Control medium—with 0.89 μM BAP: for all cultivars, the length of microshoots was 0.6 cm with a single internode. Different lowercase letters in the same column indicated the significant difference at P ≤ 0.05 (Duncan’s multiple-range test).*

**FIGURE 2:**
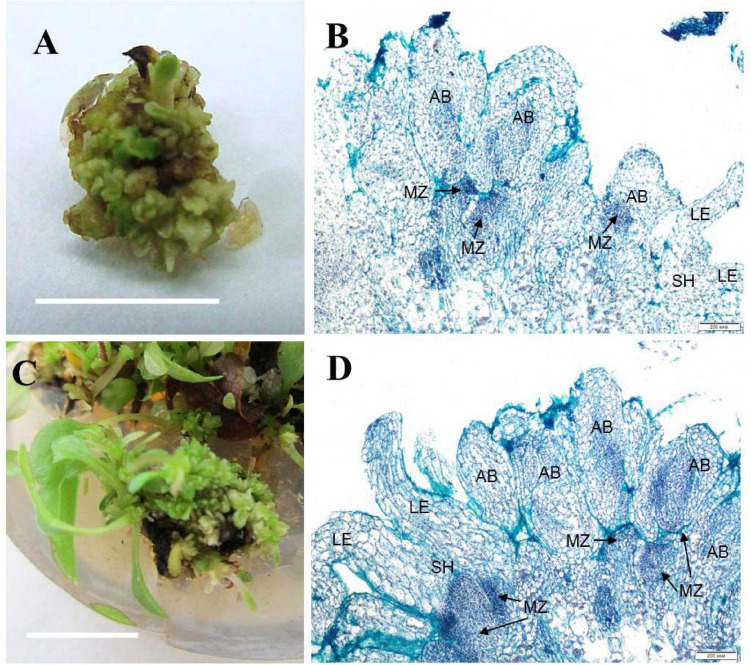
Adventitious vegetative bud regeneration via direct organogenesis of clematis plant cultivars. **(A)** Adventitious bud formation in the cultivar ‘Alpinist’ on the culture medium with 6 μM TDZ. **(B)** Meristematic tombs that developed on the surface of explants of the cultivar ‘Alpinist’ from active meristematic zones on the medium with 6 μM TDZ. Bright field. Hematoxylin and alcian blue straining. MZ—meristematic zone, AB—adventitious bud, two-leaf-stage shoot (SH—shoot, LE—leaf). **(C)** Adventitious bud and microshoot regeneration from a gemmiferous callus of the cultivar ‘Madam Julia Correvon’ on the culture medium with 6 μM TDZ. **(D)** Mass regeneration of adventitious buds and primordial leaves that developed directly on the explant surface in the cultivar ‘Madam Julia Correvon’ on the medium with 6 μM TDZ. Bright field. The section was stained with hematoxylin and alcian blue. MZ—meristematic zone, AB—adventitious bud, two-leaf-stage shoot (SH—shoot, LE—leaf). Scale bars correspond to 1 cm **(A)**, 200 μm **(B,D)**, and 0.5 cm **(C)**.

Zones of meristematic activity were mainly noted at the base of adventitious buds and microshoots, where a small amount of callus formed. Some adventitious buds regenerated directly from the determinate cells on the surface of microshoot segments. For the cultivar ‘Alpinist,’ histological analysis of the callus structure showed that it was produced by parenchymal cells, containing some inclusions, had anisotropic features, and glowed in polarized light, which is typical of starch and other polysaccharides (Figures 2A,B). Meristematically active cells were characterized by a dense cytoplasm and being large in size relative to the cell and nucleus (high nucleus/cytoplasm ratio); it was these areas that gave rise to meristems with primordial leaves. Subsequently, the development of meristems resulted in the formation of microshoots accompanied by the differentiated vascular elements connecting the apical zone of the microshoot and leaf-like structures to the main parenchymal tissue of the callus. On the periphery of the gemmiferous callus structures, small meristematic cells were evident. The callus of the cultivar ‘Madam Julia Correvon’ was produced by parenchymal tissue, the cells of which contained anisotropic inclusions. This cultivar was characterized by secondary cytodifferentiation of cells into the vascular elements that formed numerous centers of the vascular system. In the area of parenchymal cells, we also noted the presence of meristematic zones and the development of adventitious buds and microshoots (Figures 2C,D).

### Somatic Embryogenesis Induction and Plant Regeneration

#### Indirect Somatic Embryogenesis

**FIGURE 3:**
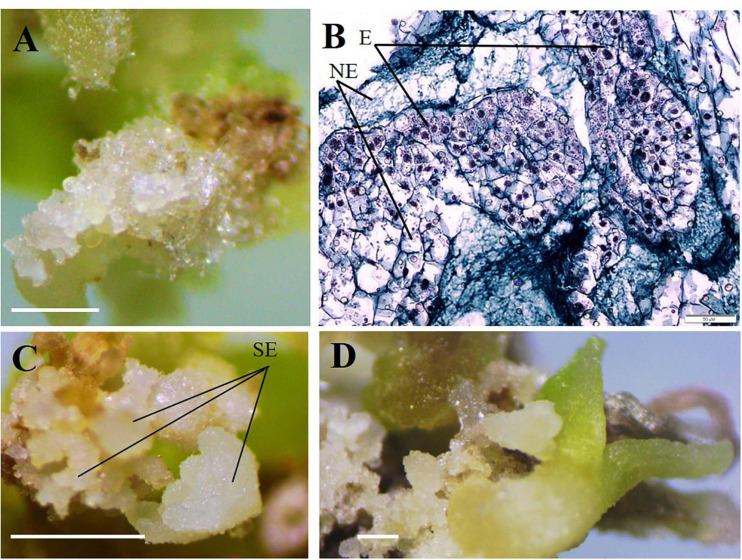
Indirect somatic embryogenesis in calli of clematis plant cultivars. **(A)** Embryogenic callus formation on the surface of vegetative buds and microshoot segments in the cultivar ‘Serenada Kryma’ on the MS medium with 2.3 μM or 4.6 μM zeatin. **(B)** Non-embryogenic (NE) and embryogenic (E) cells in the callus of the cultivar ‘Crystal Fountain,’ periclinal cell division, and somatic embryoid realization. Bright field. Methyl green–pyronin and alcian blue. **(C)** Somatic embryoids (SE) of the cultivar ‘Crystal Fountain’ at different stages of development on the medium with 1.8 μM zeatin. **(D)** Growth of somatic embryoids and the callus formed on surfaces of primary embryoids. Scale bars correspond to 1 mm **(A,C,D)** and 50 μm **(B)**.

During the microcutting of 13 cultivars with one or two internodes culturing on the MS culture medium supplemented with 1.8 μM zeatin and 0.04 μM IBA, a compact callus of light-green color formed at the base of those explants. The obtained callus was first separated from the base of the microshoots and microcuttings, then divided into segments, and transferred onto a culture medium supplemented with 2,4-D or zeatin. Table 4 reports the effects of various 2,4-D and zeatin concentrations on the induction of the embryogenic callus formation in the clematis cultivars. The presence of 2,4-D in a culture medium at the concentrations of either 4.6 μM or 6.8 μM stimulated the formation of a loosened non-embryogenic white-colored callus. In the course of this experiment, we noted that on the medium containing 1.8 μM of zeatin, cultured callus cells actively divided and, accordingly, the resulting callus had a dense structure. Embryogenic calli also formed on the media supplemented with 2.3 μM and 4.6 μM zeatin (Figure 3A). Mixed callus formation was seen to occur on the culture media containing 0.9 μM, 2.3 μM, 4.6 μM, or 6.9 μM zeatin. We should note that the appearance of meristematic zones and meristematic tubercles happened within a month’s time in this experimental work. These formations differed from the cell mass in having a bright-green color. Histological analysis revealed that two types of cells were present in the embryogenic mass: in the first, cells had a relatively dense cytoplasm, fairly thin cell walls, and very small vacuoles (embryogenic cells); the second was characterized by cells with a turbid cytoplasm and large vacuoles (non-embryogenic cells). On the 3 day of culturing, the processes of mitotic and meristematic activity in the embryogenic cell mass had become activated. Proembryo development was initiated, with asymmetric cell division occurring most often directly inside the callus (Figure 3B). Only after 12–14 days of culturing, the formation of somatic embryos from induced embryogenic determinate cells in the clematis callus was observed. Nonetheless, only during days 27–30 of culture was the embryo itself clearly visible. The presence of zeatin in the medium induced the formation of bipolar structures on the surface and inside the callus. It was possible to observe the appearance of somatic embryos on the callus surface only after 5–7 days of their formation in the callus itself (Figure 3C). All new non-zygotic embryos were light green in color and tightly connected to the maternal callus. During the cultivation of this callus structure, a part of the embryoids did separate easily, but due to the callus formed on their surface, this impeded their further development (Figure 3D).

**TABLE 4:** Callogenesis induction (means ± SE) in investigated clematis cultivars on culture medium with 2,4-dichlorophenoxyacetic acid (2,4-D) and zeatin.

Concentration of plant growth regulators (μM)	Number of explants with the callus formed (%)	Callus type (%)		
		*E*	NE	*M*
Control (0)	0^h^	0^f^	0^e^	0^d^
2,4-D				
0.9	0^h^	0^f^	0^e^	0^d^
1.8	0^h^	0^f^	0^e^	0^d^
2.3	0^h^	0^f^	0^e^	0^d^
4.6	56 ± 2.3^bc^	0^f^	100^a^	0^d^
6.8	50 ± 2.1^de^	0^f^	100^a^	0^d^
Zeatin				
0.9	20 ± 2.2^fg^	20 ± 2.6^de^	56 ± 3.3^b^	14 ± 1.2^ab^
1.8	100^a^	100^a^	0^e^	0^d^
2.3	100^a^	83 ± 6.3^ab^	6 ± 0.1^cd^	11 ± 0.7^ab^
4.6	80 ± 6.1^ab^	56 ± 3.9^ab^	14 ± 1.3^bc^	30 ± 4.6^a^
6.8	70 ± 7.2^ab^	23 ± 1.2^bc^	70 ± 4.2^ab^	7 ± 0.9^bc^

*E, embryogenic; NE, non-embryogenic; M, mixed; 0, no callus formed. Different lowercase letters in the same column indicated the significant difference at P ≤ 0.05 (Duncan’s multiple-range test).*

Table 5 presents the results of somatic embryo formation as affected by various zeatin concentrations tested. The most embryoids per explant (25) were obtained on an MS medium supplemented with 1.8 μM zeatin after 4 weeks of culture. Some primary clematis explants formed spherical structures with a diameter of 0.1–0.5 mm, yet these did not develop into full-fledged plants. Germination of somatic embryos occurred during a rather long period of cultivation (30–40 days); at first, some root growth was noted, and in the next stage, the hypocotyl had emerged and was colored green. During the somatic embryo culturing, the simultaneous germination of the shoot and root was most often observed. A typical feature of clematis seedlings was the formation and growth of two roots apparently lacking root hairs. Somatic embryoids on the culture medium with a zeatin concentration higher than 1.8 μM were not occurred. Subsequent subcultures of somatic embryos put on media with 1.8 μM zeatin combined with various concentrations of IBA led to secondary embryoids forming on the surface of already formed and developing somatic embryos (Table 6). During this experiment, it was found that, in the course of indirect somatic embryogenesis, the frequency of secondary embryogenesis depended on the IBA concentration in the culture medium. The optimal IBA concentration was 0.9 μM, in that under this condition the average number of embryoids per explant was 30, for which the average embryo size was up to 1.5 mm at the onset of the cotyledon stage. With 30 days of light exposure, the size of these cotyledons increased, to 4–5 mm. The resulting secondary embryoids easily separated from each other: their culturing showed that if the embryoid was placed in a culture vessel, it actively developed and grew, reaching 9–10 mm after 30–45 days. Nevertheless, high concentrations of IBA inhibited the embryoids’ growth and significantly reduced the frequency of secondary embryogenesis in the clematis cultivars. Further, additional somatic embryos formed, in the zone between the hypocotyl and epicotyl, on the seedlings that had developed from the primary embryoids.

**TABLE 5:** Clematis somatic embryos’ formation (means ± SE) on the MS medium with zeatin.

Cultivar*	Number of explants with formed embryoids (%)					
	Zeatin concentration (μM)					
	0.4		0.9		1.8	
A	24 ± 0.63^ab^		31 ± 0.87^ab^		89 ± 1.0^ab^	
A-N	18 ± 0.54^cd^		21 ± 0.80^ef^		73 ± 0.77^ef^	
BT	22 ± 0.52^c^		28 ± 0.82^bc^		86 ± 1.02^cd^	
CS	23 ± 0.47^ab^		29 ± 0.92^bc^		89 ± 0.87	
CF	23 ± 0.52^ab^		30 ± 1.21^ab^		86 ± 1.37^cd^	
KM	16 ± 0.58^fg^		20 ± 0.88^gh^		72 ± 0.87^g^	
LO	17 ± 0.70^ef^		21 ± 0.94^ef^		72 ± 0.86^g^	
MJC	16 ± 0.67^fg^		19 ± 0.92^i^		71 ± 1.20^gh^	
N	26 ± 0.89^a^		35 ± 0.82^a^		92 ± 0.95^a^	
NRo	15 ± 0.87^hi^		19 ± 0.77^i^		72 ± 0.95^g^	
NRu	18 ± 0.61^cd^		22 ± 0.80^cd^		75 ± 1.03^de^	
SK	25 ± 0.87^a^		30 ± 0.92^ab^		90 ± 1.04^ab^	
VZ	17 ± 0.86^ef^		20 ± 1.32^gh^		73 ± 1.15^ef^	

**Cultivar**	**Number of formed somatic embryos per explant**					
	**2 weeks**	**4 weeks**	**2 weeks**	**4 weeks**	**2 weeks**	**4 weeks**

A	0	4 ± 0.47^ab^	3 ± 0.37^a^	8 ± 0.39^a^	13 ± 0.71^a^	28 ± 0.87^a^
A-N	0	4 ± 0.37^ab^	2 ± 0.37^ab^	7 ± 0.37^ab^	10 ± 0.58^b^	24 ± 1.17^bc^
BT	0	4 ± 0.37^ab^	2 ± 0.26^ab^	7 ± 0.49^ab^	10 ± 0.47^b^	26 ± 0.70^ab^
CS	0	4 ± 0.47^ab^	3 ± 0.33^a^	7 ± 0.63^ab^	11 ± 0.42^ab^	26 ± 1.03^ab^
CF	0	4 ± 0.63^ab^	2 ± 0.30^ab^	7 ± 0.37^ab^	9 ± 0.39^bc^	27 ± 0.70^ab^
KM	0	4 ± 0.45^ab^	2 ± 0.37^ab^	6 ± 0.33^bc^	8 ± 0.76^d^	22 ± 0.67^cd^
LO	0	4 ± 0.56^ab^	1 ± 0.21^bc^	7 ± 0.54^ab^	9 ± 0.56^bc^	22 ± 0.87^cd^
MJC	0	3 ± 0.58^bc^	1 ± 0.15^bc^	6 ± 0.42^bc^	9 ± 0.58^bc^	23 ± 1.04^bc^
N	0	5 ± 0.39^a^	3 ± 0.37^a^	8 ± 0.39^a^	13 ± 0.87^a^	29 ± 0.98^a^
NRo	0	4 ± 0.52^ab^	1 ± 0.30^bc^	7 ± 0.56^ab^	9 ± 0.37^bc^	24 ± 1.26^bc^
NRu	0	4 ± 0.52^ab^	2 ± 0.30^ab^	6 ± 0.45^bc^	9 ± 0.52^bc^	24 ± 0.93^bc^
SK	0	5 ± 0.76^a^	2 ± 0.30^ab^	8 ± 0.37^a^	12 ± 0.63^ab^	27 ± 0.61^ab^
VZ	0	3 ± 0.60^bc^	2 ± 0.30^ab^	7 ± 0.37^ab^	8 ± 0.67^d^	23 ± 0.52^bc^

*A, ‘Alpinist’; A-N, ‘Ay-Nor’; BT, ‘Bal Tsvetov’; CS, ‘Crimson Star’; CF, ‘Crystal Fountain’; KM, ‘Kosmicheskaya Melodiya’; LO, ‘Lesnaya Opera’; MJC, ‘Madame Julia Correvon’; N, ‘Nevesta’; NRo, ‘Nikitsky Rosovyi’; NRu, ‘Nikolay Rubtsov’; SK, ‘Serenada Kryma’; VZ, ‘Vechniy Zov.’ Control medium—free plant growth regulator medium. Different lowercase letters in the same column indicated the significant difference at P ≤ 0.05 (Duncan’s multiple-range test).

**TABLE 6:** Indole-3-butyric acid (IBA) concentration influence on the frequency of secondary embryogenesis (means ± SE) in investigated clematis cultivars cultured on the MS medium with 1.8 μM zeatin.

Cultivar*	Number of explants with secondary embryoids formed (%)							
	IBA concentration (μM)							
	0.4		0.9		1.8		4.6	
A	36 ± 0.94^ab^		99 ± 0.42^ab^		30 ± 0.88^ab^		10 ± 0.52^b^	
A-N	26 ± 0.79 ^cd^		90 ± 0.83^e^		28 ± 0.87^bc^		8 ± 0.49^c^	
BT	36 ± 0.92^ab^		98 ± 0.52^ab^		30 ± 1.01^ab^		10 ± 0.52^b^	
CS	43 ± 0.73^ab^		100^a^		32 ± 0.77^a^		11 ± 0.63^ab^	
CF	33 ± 0.99^ab^		98 ± 0.84^ab^		32 ± 0.95^a^		11 ± 0.71^ab^	
KM	28 ± 1.12^bc^		92 ± 1.05^bc^		27 ± 0.65^cd^		7 ± 0.58^de^	
LO	29 ± 0.89^b^		90 ± 0.97^e^		25 ± 0.68		7 ± 0.37^de^	
MJC	25 ± 1.0^ef^		92 ± 0.91^bc^		27 ± 0.83^cd^		8 ± 0.39^c^	
N	46 ± 1.15^a^		100^a^		32 ± 0.58^a^		13 ± 0.77^a^	
NRo	23 ± 0.99^gh^		91 ± 0.82^cd^		28 ± 0.82^bc^		9 ± 0.58^bc^	
NRu	30 ± 1.17^ab^		92 ± 1.32^bc^		29 ± 0.98^ab^		9 ± 0.73^bc^	
SK	46 ± 1.04^a^		100^a^		30 ± 0.86^ab^		12 ± 0.67^ab^	
VZ	27 ± 1.28^c^		93 ± 1.31^b^		27 ± 0.58^cd^		9 ± 0.52^bc^	

**Cultivar**	**Number of secondary embryoids per explant**							
	**2 weeks**	**4 weeks**	**2 weeks**	**4 weeks**	**2 weeks**	**4 weeks**	**2 weeks**	**4 weeks**

A	3 ± 0.39^c^	14 ± 0.63^ab^	18 ± 0.82^a^	30 ± 0.77^ab^	2 ± 0.33^bc^	5 ± 0.33^b^	0	3 ± 0.30^ab^
A-N	2 ± 0.33^de^	9 ± 0.52^de^	13 ± 0.56^cd^	33 ± 0.73^ab^	4 ± 0.52^a^	6 ± 0.37^ab^	0	2 ± 0.21^b^
BT	3 ± 0.26^c^	13 ± 0.54^ab^	17 ± 0.83^ab^	34 ± 0.88^ab^	2 ± 0.42^bc^	5 ± 0.63^b^	0	2 ± 0.21^b^
CS	4 ± 0.54^b^	14 ± 0.60^ab^	17 ± 0.37^ab^	33 ± 0.84^ab^	3 ± 0.45^ab^	5 ± 0.37^b^	0	2 ± 0.42^b^
CF	4 ± 0.37^b^	14 ± 0.73^ab^	18 ± 0.68^a^	34 ± 0.63^ab^	4 ± 0.52^a^	6 ± 0.52^ab^	0	4 ± 0.52^a^
KM	2 ± 0.39^de^	9 ± 0.63^de^	12 ± 0.98^ef^	28 ± 0.70^bc^	2 ± 0.33^bc^	5 ± 0.37^b^	0	2 ± 0.33^b^
LO	2 ± 0.45^de^	9 ± 0.61^de^	13 ± 0.88^cd^	25 ± 0.83^ef^	0^d^	4 ± 0.33^cd^	0	2 ± 0.37^b^
MJC	2 ± 0.37^de^	11 ± 0.58^bc^	12 ± 0.82^ef^	26 ± 0.58^d^	0^d^	4 ± 0.42^cd^	0	0^c^
N	6 ± 0.52^a^	18 ± 0.65^a^	18 ± 0.58^a^	36 ± 0.73^a^	4 ± 0.47^a^	7 ± 0.37a	0	4 ± 0.30^a^
NRo	2 ± 0.39^de^	11 ± 0.60^bc^	13 ± 0.76^cd^	26 ± 0.49^d^	0^d^	4 ± 0.37^cd^	0	0^c^
NRu	2 ± 0.26^de^	11 ± 0.54^bc^	14 ± 0.70^cd^	27 ± 0.61^c^	2 ± 0.37^bc^	4 ± 0.21^cd^	0	0^c^
SK	5 ± 0.49^ab^	12 ± 0.67^b^	17 ± 0.63^ab^	33 ± 0.75^ab^	3 ± 0.37^ab^	6 ± 0.37^ab^	0	3 ± 0.33^ab^
VZ	2 ± 0.30^de^	9 ± 0.42^de^	13 ± 0.89^cd^	25 ± 0.47^ef^	0^d^	4 ± 0.42^cd^	0	2 ± 0.37^b^

*A, ‘Alpinist’; A-N, ‘Ay-Nor’; BT, ‘Bal Tsvetov’; CS, ‘Crimson Star’; CF, ‘Crystal Fountain’; KM, ‘Kosmicheskaya Melodiya’; LO, ‘Lesnaya Opera’; MJC, ‘Madame Julia Correvon’; N, ‘Nevesta’; NRo, ‘Nikitsky Rosovyi’; NRu, ‘Nikolay Rubtsov’; SK, ‘Serenada Kryma’; VZ, ‘Vechniy Zov.’ Control medium—culture medium with 1.8 μM zeatin. Different lowercase letters in the same column indicated the significant difference at P ≤ 0.05 (Duncan’s multiple-range test).

**FIGURE 4:**
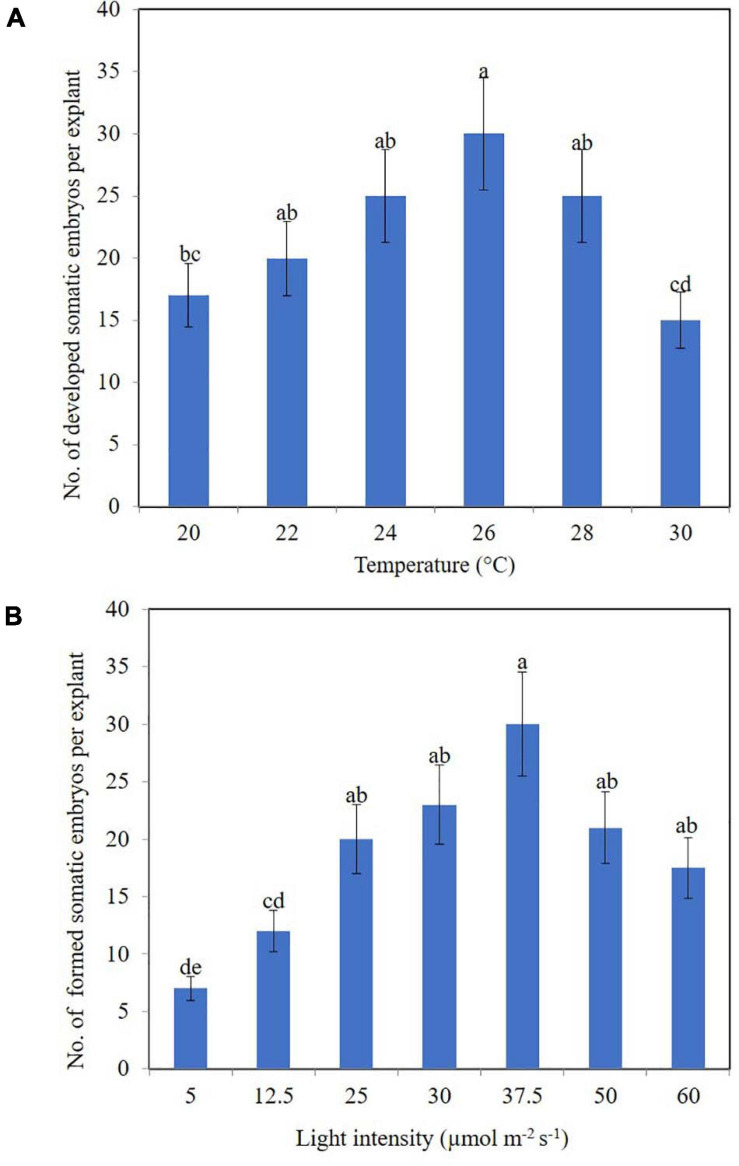
Somatic embryoids’ regeneration of clematis plant cultivars on the medium with 1.8 μM zeatin under different culturing conditions: temperature **(A)** and light intensity **(B)**. Bars represent the mean ± SE (*n* = 3). Bars marked with different letters indicate statistically different values between the realized morphogenic capacity of explants via somatic embryogenesis, at different temperatures and light intensities, according to Duncan’s test (*P* ≤ 0.05). The experiments were performed three times.

Our research also uncovered the effects of physical factors upon the development of somatic embryos in the calli of clematis. In this case, a drop or rise in temperature significantly influenced the number of developed somatic embryoids (Figure 4A), with the best results for development somatic embryoid obtained at 26°C, when its numbers reached 30 units per explant. Among the embryos, however, embryoids at different stages of their development could be observed, spanning the globular to cotyledon phase.

Unlike temperature, light intensity did have a significant effect upon the number of formed somatic embryoids. Reducing the light level to 12.5 μmol m^–2^ s^–1^ strongly reduced the number of somatic embryos formation (Figure 4B). Optimal light intensity was determined to be 37.5 μmol m^–2^ s^–1^. Under this condition, the number of formed embryoids reached 25–30 per explant. All the somatic embryos were bright green in color. Additionally, 80% of explants cultured at this light intensity level had competent cells; that is, they were capable of forming non-zygotic embryos.

#### Direct Somatic Embryogenesis

**FIGURE 5:**
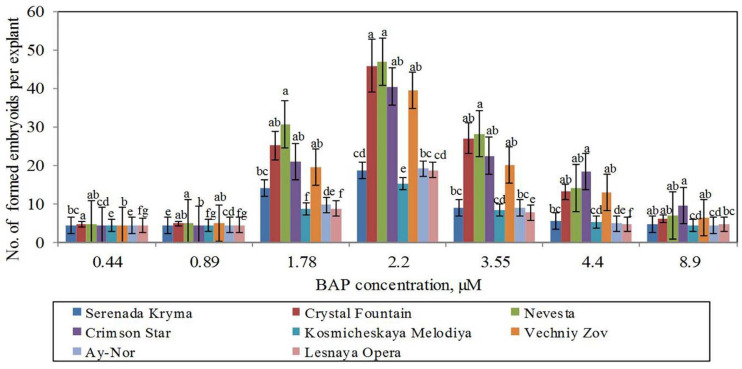
Somatic embryoids’ formation in eight cultivars of clematis plants on the MS culture medium containing different BAP concentrations with 0.09 μM IBA. Bars represent the mean ± SE (*n* = 3), for which different letters represent a significant difference, according to Duncan’s test (*P* ≤ 0.05).

**FIGURE 6:**
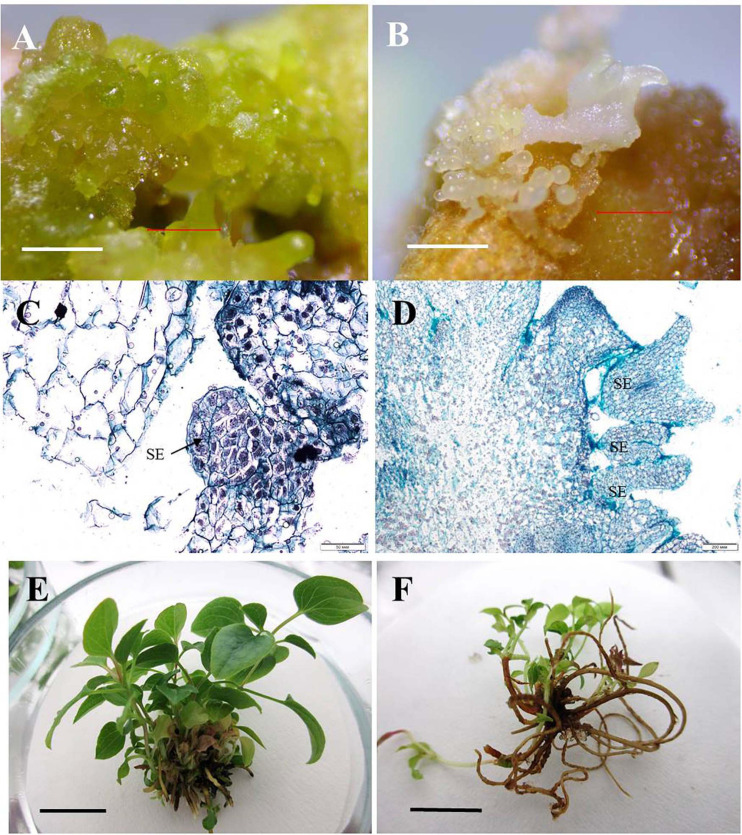
Direct somatic embryogenesis in clematis plant cultivars. **(A)** Globular structures on the surface of a vegetative bud in the cultivar ‘Nevesta’ on the MS culture medium, with 2.2 μM BAP and 0.09 μM IBA, after 20 days of somatic embryogenesis induction. **(B)** Embryoids on the globular and torpedo growth stages of the cultivar ‘Crystal Fountain’ on the medium with 2.2 μM BAP and 0.09 μM IBA. **(C)** Meristematic active zone and globular embryoid formation in the cultivar ‘Nevesta.’ Bright field. Methyl green–pyronin and alcian blue. SE—somatic embryo. **(D)** Somatic embryoids of the cultivar ‘Crystal Fountain’ at different stages of its development. Bright field. The section was stained with hematoxylin and alcian blue. SE—somatic embryo. **(E)** Plantlets’ development from somatic embryoids of the cultivar ‘Nevesta.’ **(F)** Developed plantlets from somatic embryoids of the cultivar ‘Crystal Fountain.’ Scale bars correspond to 1 mm **(A,B)**, 50 μm **(C)**, 200 μm **(D)**, and 1 cm **(E,F)**.

For the induction of direct somatic embryogenesis, BAP was used in various concentrations (0.44–8.9 μM) in the combination with a fixed IBA concentration (0.09 μM). Through this experimental work, firstly, we were able to induce direct somatic embryogenesis in all 13 studied clematis cultivars, but a genotypic influence was discernable: at 30 days since the buds were placed on media for culturing, the number of somatic embryos formed was different for each cultivar *in vitro*. At the base of the buds in ‘Alpinist,’ ‘Bal Tsvetov,’ ‘Madame Julia Correvon,’ ‘Nikitsky Rosovyi,’ and ‘Serenada Kryma’ cultivars, only single somatic embryos were formed. Secondly, we found that the ability of explants to form embryoids depended on the BAP concentrations. The optimal BAP concentration was 2.20 μM, in that this promoted the induction of somatic embryogenesis and the formation of the maximum number of embryos in ‘Ay-Nor,’ ‘Crimson Star,’ ‘Crystal Fountain,’ ‘Kosmicheskaya Melodiya,’ ‘Lesnaya Opera,’ ‘Nevesta,’ ‘Serenada Kryma,’ and ‘Vechniy Zov’ cultivars (Figure 5). In the control medium lacking any plant growth regulators, the development of microshoots was observed occasionally in some cultivars (‘Crystal Fountain,’ ‘Crimson Star,’ ‘Kosmicheskaya Melodiya,’ ‘Lesnaya Opera’). Increasing the BAP concentration to 4.40 μM also promoted shoot formation, rather than the formation of embryoids; however, far fewer microshoots emerged than when cultured on a medium supplemented with 2.20 μM of BAP. Moreover, we noted that the buds of clematis cultivars from the groups Lanuginosa and Florida displayed a higher morphogenetic capacity. The number of somatic embryos in ‘Nevesta’ cultivar was ca. 40 per explant. The formation of somatic embryos always took place directly on the surface of vegetative buds, most often in the zone of apex meristematic cells (Figure 6A). After 20 days of culturing, the formation of multiple globular structures was evident; they were absolutely smooth, round, or slightly oblong in shape, and light green and yellow in color. Initially, these structures lay very close to each other (Figures 6B,C). In this form, at the same time they could be up to the heart, torpedo, or cotyledon stages. After 30–40 days of culturing, the embryoids began to separate from each other somewhat easily (Figure 6D), but the extent to which this process occurred depended on the clematis cultivar. The somatic embryos of ‘Crystal Fountain,’ ‘Nevesta,’ ‘Crimson Star,’ and ‘Ay-Nor’ were those that best separated from each other. A typical feature of embryoids during this period was root germination, after which regeneration of the microshoot began (Figures 6C,D). The average root and shoot length after 14 days of germination did not significantly differ among the cultivars. After germination and 21 days of culturing, the roots became actively developed in the cultivars ‘Nevesta,’ ‘Crimson Star,’ ‘Vechniy Zov,’ and ‘Ay-Nor,’ whose average lengths were 4.8, 4.5, 4.2, and 3.9 cm, respectively. Further, seedlings of the cultivars ‘Nevesta,’ ‘Crimson Star,’ and ‘Ay-Nor’ formed between 2.1 and 2.5 roots per explant.

The frequency of secondary embryogenesis varied across the studied cultivars. A high embryogenesis rate, upward of 65%–100%, was observed in the cultivars ‘Nevesta,’ ‘Crimson Star,’ ‘Serenada Kryma,’ ‘Crystal Fountain,’ and ‘Ay-Nor’ when cultured on the medium with 2.20 μM of BAP and 0.09 μM of IBA (Table 7). The three other cultivars showed a low frequency of secondary embryogenesis, which did not exceed 30%–50%. Induction of embryoid formation, development, and plant regeneration occurred on the same culture medium. When somatic embryos selected from the globular, heart, and torpedo stages were placed on media with a reduced BAP content or free of cytokinin, the embryoid’s development halted and could go 2–3 weeks without any signs of growth. Thus, these embryos could be grouped by size and stage of development, for later propagation in a biotechnological system, if necessary.

**TABLE 7:** Secondary embryogenesis (means ± SE) in eight cultivars of clematis during primary somatic embryos culture on the MS medium with 6-benzylaminopurine (BAP) with 0.09 μM IBA.

Cultivar	Frequency of secondary embryogenesis (%)	
	BAP concentration (μM)	
	0.89	2.22
Serenada Kryma	15 ± 1.3^bc^	65 ± 4.5^b^
Crystal Fountain	53 ± 3.4^a^	100^a^
Nevesta	26 ± 2.2^ab^	97 ± 4.3^a^
Crimson Star	21 ± 1.6^ab^	100^a^
Kosmicheskaya Melodiya	0^d^	35 ± 3.1^de^
Vechniy Zov	0^d^	57 ± 3.9^bc^
Ay-Nor	26 ± 3.3^ab^	92 ± 4.2^ab^
Lesnaya Opera	0^d^	32 ± 1.5^e^

*Control medium—free plant growth regulator medium. Different lowercase letters in the same column indicated the significant difference at P ≤ 0.05 (Duncan’s multiple-range test).*

Secondary embryos most often formed along the edges of the somatic embryoids’ cotyledons. Nonetheless, embryo formation directly from the apical zone of embryoids was also noted. Initially, the formation of transparent round structures was observed, but then they became white and increased in size (Figure 6E). Since this process occurred in light, globular embryoids were stained green and had a smooth shiny surface. Though the embryos were positioned tightly adjacent to each other, they could easily be divided among themselves. The ensuing plantlets developed and formed the cultivar-specific plants (Figure 6F), whose overall frequency was 80%–95%, on average.

## Discussion

### Organogenesis and Somatic Embryogenesis Are the Main Plant Regeneration Modes of Clematis

The widespread use of biotechnological research in the introduction, breeding, and propagation of plants has enabled us to significantly advance our knowledge of plant biology and actively use them in studying morphogenesis via somatic embryogenesis and organogenesis. It is well known that exogenous factors significantly affect the realized morphogenetic capacity of plant tissues and organs; hence, only by combining the results from multiple experiments can an efficient system of somatic embryogenesis and organogenesis for a specific crop be developed that would work in most plant species and cultivars. Most of the clematis plants that housed the NBG-NSC open-field collection are old-aged (more than 170 years old). Studies of the morphogenetic capacity of tissues and organs in mature plants *in vitro* are of great scientific and practical interest. Firstly, this is because using mature plant tissue as a primary explant allows one to obtain plant material with economically valuable features. Yet working with mature plant tissues is hampered by the fact that as the plant ages, the processes that result in growth inhibition and reduced regenerative ability also occur in the plant tissues and organs. Microshoots obtained via regeneration are thus characterized by slow growth and weak rooting ability.

Despite this obstacle, plant scientists all over the world continue to work on this problem, pursuing their research programs and developing more and more biotechnologies applicable to specific crop (Geneve and Preece, 1997; Jain and Ishii, 2003; Preil, 2003; Rout and Jain, 2004; Rout et al., 2006; Mitrofanova, 2011; Germanà and Lambardi, 2016; Mitrofanova et al., 2019; Narvaez et al., 2019). For our study here, we selected 13 cultivars from the main clematis groups that demonstrated different morphogenetic capacities.

It is known that plant growth regulators play an essential role in the interaction of plant cells, tissues, and organs. Low concentrations of these substances are necessary for the induction and regulation of key physiological and morphogenic processes. For each plant species and cultivar, the optimal concentrations and combinations of plant growth regulators in the culture medium could be experimentally deduced. Thus, cytokinins, in addition to enhancing cell division and growth processes, stimulate cell differentiation, histogenesis, and shoot formation; they also affect callus differentiation and induce the development of axillary buds, the growth of lateral shoots, adventitious bud formation, and subsequent regeneration of plants (Kalinin et al., 1992; Kunah, 2005; Mitrofanova, 2011; Loyola-Vargas and Ochoa-Alejo, 2018). In our present work, zeatin, BAP, and TDZ were used as the main cytokinins in tissue and organ culturing of clematis plants. Along with them, the auxins 2,4-D, NAA, and IBA were used to induce the processes of organogenesis and somatic embryogenesis. Each of them performed its function as expected. Auxins, activating the process of cell division and stretching, are required for the formation of vascular and root systems in plants (Horvath et al., 2006; Novikova et al., 2013; Kuluev et al., 2015). Auxin-saturated tissues have an attracting effect, that is, the ability to attract nutrients, which are then deposited as reserves in seeds, fruits, tubers, and root crops or are instead actively used during the growth and development of the meristem. Auxins play a crucial role in cell differentiation. For example, the induction of division in resting vacuolated parenchymal cells from an auxin application is dedifferentiation. Almost immediately, auxin causes the phenomenon of apical dominance by inhibition of axillary soot growth by the continued meristematic activity in shoot apex. The auxin–cytokinin interaction regulates the coordination of the main and lateral shoot formation and growth (Bari and Jones, 2009; Davies, 2010; Fatima and Anis, 2012; Rademacher, 2015; Bhatla, 2018; Yegorova et al., 2019).

Theoretical studies in the field of somatic embryogenesis have shown that the whole embryogenic cell mass is determined by the process of embryoid formation, but this is not entirely true. Only some cells are capable of forming a somatic embryo. Observations of clover and pistachios support the hypothesis that growth regulators initiate asymmetric division and lead to a change in cell polarity (Maheswaran and Williams, 1985; Onay, 2000). It is likely that exogenous plant growth regulators directly change the polarity of the cells by interfering with the pH gradient or the electric field around the cells (Mitrofanova, 2011; Germanà and Lambardi, 2016). In our previously histological analysis of clematis cultivars, we noted the appearance of two distinct cell types: deterministic and non-deterministic (Mitrofanova, 2011). Observations during this experiments revealed no less than seven morphological types of new somatic embryos capable of forming in clematis plants: (1) monocotyledonous, in which the embryoid is formed from one cotyledon, and the second, most often, is underdeveloped or completely reduced; (2) dicotyledonous, which resembles a zygotic embryo in clematis; (3) polycotyledonous, meaning a newly developing embryoid having three or more cotyledons; (4) tube-like, in which the embryoid cotyledons inosculate in the form of a tube; (5) an embryoid with an elongated hypocotyl and almost reduced cotyledons (the cotyledons of the embryo are very narrow and the apex is not clear in the epicotyl zone); (6) an embryoid similar to a zygotic embryo, thus resembling a zygotic embryo, yet in which development stops at the stage of cotyledon opening, after which the embryoid dies; and (7) an embryoid in the form of an open terry bud, the cotyledons of which grow vigorously and deformed. During the subsequent culturing of all types of somatic embryos and embryo-like structures, we found that only the first four morphological types of embryoid featured the capacity to regenerate plants (Mitrofanova, 2011).

There is a close correlation between the effect of light quality on a plant and the accumulation of certain hormones and growth inhibitors in it. We also know that the optimum temperature for culturing somatic embryos of most plant species is in the range of 21–25°C (Merkle, 1997; Han and Park, 1999; Jain and Ishii, 2003; Jain and Gupta, 2018). A temperature of 28°C stimulated the induction of somatic embryogenesis in *Pinus halepensis* Miller (Pereira et al., 2016), whereas we detected a high number of developed somatic embryos in clematis cultivars at a lower temperature of 26°C. Our results for how light intensity influenced the production of clematis somatic embryos differ markedly from those obtained in other crops, since those embryoids are usually formed in the dark (Oliveira and Pais, 1992; Mitrofanova et al., 1997; Dal Vesco and Guerra, 2001; Germanà and Lambardi, 2016; Corredoira et al., 2019). This once again confirms the need for thorough studies of the regeneration features and selection of suitable culture conditions for each new species or plant cultivar.

Direct somatic embryogenesis is the process of somatic embryoid formation directly from cells of somatic explant tissues *in vitro* and is thus more similar to the formation of zygotic embryos. This plant regeneration pathway was first described 40 years ago (Sharp et al., 1980). The somatic development of the embryos is very flexible, as it is influenced by both the plant genotype itself and the culture conditions applied to it. Since then, the main parameters determining somatic embryogenesis have been elucidated, namely, being the type of explant, the stage of the explant’s development, and interactions between the culturing conditions and the explant (Souter and Lindsey, 2000; Hamama et al., 2001; Nanda and Rout, 2003; Correia et al., 2011; Zhang et al., 2011; Nic-Can et al., 2015; Germanà and Lambardi, 2016; Jain and Gupta, 2018; Loyola-Vargas and Ochoa-Alejo, 2018; Corredoira et al., 2019; Mitrofanova et al., 2019).

We had noted that there was a zone of activation of somatic embryo formation; that is, a particular clematis explant of eight cultivars (‘Ay-Nor,’ ‘Crimson Star,’ ‘Crystal Fountain,’ ‘Kosmicheskaya Melodiya,’ ‘Lesnaya Opera,’ ‘Nevesta,’ ‘Serenada Kryma,’ ‘Vechniy Zov’) was able to induce somatic embryogenesis, both primary and secondary. This zone of somatic embryogenesis induction (“inducer”) in culture vessels could be one or a group of somatic embryos. Perhaps, this driver is the center of *in vitro* plant embryogenesis. It is known that in animals, the reacting system, which undergoes differentiation under the influence of an “inductor,” often becomes an inducer for new organs and tissues, and the entire development of the embryo can thus be viewed as a chain of successive induction interactions (Souter and Lindsey, 2000; Fehér and Magyar, 2015). A similar process was observed here in the studied clematis plants. It may be surmised that the “inducer’s” effect on neighboring explants manifests via the culture medium into which inducing substances (metabolites and hormones, among others) are released, or, perhaps, this effect comes from the altered electric fields transmitted directly from the explant to the explant within the culture vessel. In our experiments, using the medium on which the “inducer” was cultured to activate the somatic embryogenesis in clematis was not successful. Accordingly, we could rule out the influence from the “inducer” through the culture medium *per se*. Possibly, the action through the medium is short-term in nature and we were not able to fixate this moment. Furthermore, to implement this induction, it is necessary that the cells exposed to the “inducer” have the appropriate competence. When it comes to a biotechnological *in vitro* propagation system, only competent cells are involved in the plant regeneration. In addition, the determination of induced clematis cells occurred after 7–8 days of culturing. Published data on the prevalence of embryogenic capacity in cultured plant cells indicates that, among 10^3^–10^4^ cells, just one will have the ability to form a somatic embryo (Hari, 1980). On a suspension culture of carrots, an original method was applied, involving the fractionation of the initial cell suspensions and isolation of cell fractions that was characterized by high embryogenic capacity (Fujimura and Komanima, 1979). It is known that applying fractionation methods makes it possible to obtain fractions of single globular, heart-shaped, and torpedo-shaped embryoids and plantlets in preparative amounts (Moiseeva, 1991). In our experiments, the effect of the “inducer” on somatic embryogenesis, expressed in the active formation of adventitious embryos on explants placed on a culture medium, was observed in almost all the clematis cultivars except for ‘Kosmicheskaya Melodiya.’ For this cultivar, perhaps we were unable to identify such an activation zone for its embryogenic processes. Removal of the “inducer” from the culture vessel significantly reduced the frequency of embryogenesis, or regeneration did not occur at all. Further, it was evident that the “inducer” can operate for a sufficiently long period of time. In some cultivars—‘Crystal Fountain,’ ‘Nevesta,’ and ‘Crimson Star’—this phenomenon was observed for 2–3 years (unpublished data). Thus, for both primary and secondary somatic embryogenesis, the biotechnological system was the most effective in the clematis groups Lanuginosa and Florida.

Despite available reports on the critical period in the process of *in vitro* plant regeneration and the need to change culture media at various stages of morphogenesis (Merkle, 1997; Zhang et al., 2011; Germanà and Lambardi, 2016; Jain and Gupta, 2018; Loyola-Vargas and Ochoa-Alejo, 2018; Tomiczak et al., 2019), in the studied clematis cultivars we found no evidence of cell addiction to exogenous plant growth regulators and a delayed somatic embryogenesis process. In our opinion, the somatic embryo or group of somatic embryos, temperature, and light intensity are the critical factors for stimulating or inhibiting the process of proembryo formation.

In conclusion, based on our study’s results, we were able to reveal that further development of somatic embryoids and obtaining full-fledged regenerated plantlets of clematis cultivars were successful under the same conditions as the induction of somatic embryogenesis. Unlike many other plant species, in whose propagation via somatic embryogenesis two or three culture media are sequentially used (Correia et al., 2011; Corredoira et al., 2015; Jain and Gupta, 2018), our clematis somatic embryos and regenerants from them were obtained on the same medium. An interesting fact was that BAP was used effectively as an exogenous cytokinin to induce the direct regeneration of somatic embryos. It is known that in most plants cultured *in vitro*, the auxin 2.4-D is typically used for this goal (Raghavan, 2004; Mitrofanova, 2011; Correia et al., 2012; de Alcantara et al., 2014; Germanà and Lambardi, 2016; Jain and Gupta, 2018). The next stage of investigation is to discover the molecular mechanisms underpinning the realization of morphogenetic potential, especially the induction of somatic embryogenesis, the development and death of somatic embryos, and plant formation (Smertenko and Bozhkov, 2014; Fehér, 2019; Méndez-Hernández et al., 2019).

## Conclusion

In this comprehensive study, both organogenesis and somatic embryogenesis were induced from the base of clematis vegetative buds and microshoots. An increase in regenerative capacity by adventitious shoot formation for 13 cultivars due to TDZ in the culture medium was demonstrated. The main factors affecting the process of somatic embryo regeneration were established: these were mother plant genotype, plant growth regulators (BAP and IBA), a temperature of 26°C, and a light intensity of 37.5 μmol m^–2^ s^–1^. In the case of indirect somatic embryogenesis as well as its direct one, secondary embryogenesis was always observed, which significantly increased the frequency of somatic embryo formation and plant regeneration for eight cultivars. As far as we know, this is the first time that the studied clematis cultivars have been shown to possess high morphogenic capacity due to a combination of methods applied during their plant micropropagation. In so doing, this work paves the way for the letting use biotechnology tools, such as cryobank creation, genomic investigation, and genetic transformation (among others). The presented system of somatic embryogenesis allows not only for the propagation of clematis species and cultivars but also to save plant material in the form of slow-growing *in vitro* collections which could be used to replenish collections of other botanical gardens.

## Data Availability Statement

The raw data supporting the conclusions of this article will be made available by the authors, without undue reservation.

## Author Contributions

NZ provided the clematis cultivars for the experiments and assigned them to groups. NI and OM were responsible for the *in vitro* experiments. TK prepared the permanent slides for microscopy, and TK and IM described and analyzed them. IM and OM checked and corrected the manuscript. IM planned this research and its experiments and wrote the manuscript. All coauthors agreed with the content of the manuscript and support its conclusions.

## Conflict of Interest

The authors declare that the research was conducted in the absence of any commercial or financial relationships that could be construed as a potential conflict of interest.
