# Supplementary material for: In vitro Regeneration of Clematis Plants in the Nikita Botanical Garden via Somatic Embryogenesis and Organogenesis
Source: Front Plant Sci. 2021 Mar 12;12:541171. doi: 10.3389/fpls.2021.541171 (PMC7994861; doi:10.3389/fpls.2021.541171)
Supplement: Supplementary file 1 [file Data_Sheet_1.PDF]

ANOVA TABLE 2 (cultivar and agent effects on average number of regenerated microshoots per explant)

Effect	SS	DF	MS	F	P
Cultivar	50.5228	12	4.2102	1.8350	0.93
BAP or TDZ	150.3704	1	150.3704	65.5392	1.00
Cultivar * BAP or TDZ	29.354	12	2.4462	1.0662	0.59
Error	119.3066	23	2.2943		

Significant at $p < 0.05$

ANOVA TABLE 2 (cultivar and concentration effects on average number of regenerated microshoots per explant)

Effect	SS	DF	MS	F	P
Cultivar	50.5228	12	4.2102	0.7939	0.35
BAP or TDZ concentration	85.6172	6	42.8086	8.0726	1.00
Cultivar * BAP or TDZ concentration	6.5995	72	0.2750	0.0519	0.00
Error	206,815	90	5,3029		

Significant at $p < 0.05$

ANOVA TABLE 2 (concentration and agent effects on average number of regenerated microshoots per explant)

Effect	SS	DF	MS	F	P
Concentration	85.6172	6	42.8086	33.3041	1.00
BAP or TDZ	150.3704	1	150.3704	65.5392	1.00
Concentration * BAP or TDZ	21.0192	6	10.5096	8.1762	1.00
Error	92.5476	72	1.2853		

Significant at $p < 0.05$

ANOVA TABLE 3 (Length of explant)

Effect	SS	DF	MS	F	p
BAP or TDZ concentration	6.801	6	3.400	391.23	0.0000
Cultivar	0.829	12	0.069	7.96	0.0000
BAP or TDZ concentration * Cultivar	0.168	72	0.007	0.81	0.7090
Error	0.3390	39	0.932		

Significant at $p < 0.05$

ANOVA TABLE 3 (Number of internodes)

Effect	SS	DF	MS	F	p
BAP or TDZ concentration	24.659	6	12.329	87.429	0.0000
Cultivar	3.898	12	0.324	2.304	0.0246
BAP or TDZ concentration * Cultivar	4.713	72	0.196	1.393	0.1749
Error	5.500	39	0.141		

Significant at $p < 0.05$
